# A qualitative study of physician perceptions and experiences of caring for critically ill patients in the context of resource strain during the first wave of the COVID-19 pandemic

**DOI:** 10.1186/s12913-021-06393-5

**Published:** 2021-04-22

**Authors:** Jeanna Parsons Leigh, Laryssa G. Kemp, Chloe de Grood, Rebecca Brundin-Mather, Henry T. Stelfox, Josh S. Ng-Kamstra, Kirsten M. Fiest

**Affiliations:** 1 Faculty of Health, School of Health Administration, Dalhousie University, Halifax, Nova Scotia Canada; 2 Department of Critical Care Medicine, University of Calgary, Calgary, Alberta Canada; 3 Department of Community Health Sciences, University of Calgary, Calgary, Alberta Canada; 4 O’Brien Institute for Public Health, University of Calgary, Calgary, Alberta Canada

**Keywords:** Qualitative research, Resource strain, COVID-19, Critical care physicians

## Abstract

**Background:**

The COVID-19 pandemic has led to global shortages in the resources required to care for critically ill patients and to protect frontline healthcare providers. This study investigated physicians’ perceptions and experiences of caring for critically ill patients in the context of actual or anticipated resource strain during the COVID-19 pandemic, and explored implications for the healthcare workforce and the delivery of patient care.

**Methods:**

We recruited a diverse sample of critical care physicians from 13 Canadian Universities with adult critical care training programs. We conducted semi-structured telephone interviews between March 25–June 25, 2020 and used qualitative thematic analysis to derive primary themes and subthemes.

**Results:**

Fifteen participants (eight female, seven male; median age = 40) from 14 different intensive care units described three overarching themes related to physicians’ perceptions and experiences of caring for critically ill patients during the pandemic: 1) Conditions contributing to resource strain (e.g., continuously evolving pandemic conditions); 2) Implications of resource strain on critical care physicians personally (e.g., safety concerns) and professionally (e.g. practice change); and 3) Enablers of resource sufficiency (e.g., adequate human resources).

**Conclusions:**

The COVID-19 pandemic has required health systems and healthcare providers to continuously adapt to rapidly evolving circumstances. Participants’ uncertainty about whether their unit’s planning and resources would be sufficient to ensure the delivery of high quality patient care throughout the pandemic, coupled with fear and anxiety over personal and familial transmission, indicate the need for a unified systemic pandemic response plan for future infectious disease outbreaks.

**Supplementary Information:**

The online version contains supplementary material available at 10.1186/s12913-021-06393-5.

## Background

Concerns about the physical and psychological consequences for healthcare providers tasked with caring for patients with the novel-coronavirus SARS-CoV-2, the virus causing COVID-19, have been well documented [1–5]. From early in the pandemic, reports from countries around the world have detailed experiences of increased emotional distress, symptoms of depression, anxiety, insomnia and overall mental health disturbances among frontline staff [6–11]. Furthermore, retrospective studies following the severe acute respiratory syndrome (SARS) outbreak in 2003 have shown that some healthcare providers who worked in locations where contact with SARS patients was common continue to experience symptoms of posttraumatic stress disorder years later [12–14]. This is especially true in instances where psychological supports were minimal at the time [15]. Reduced confidence in training, fear of the unknown, limited hospital capacity, and insufficient support of personal protective equipment, are just some of the factors that have been shown to heighten healthcare worker experiences of negative psychological symptoms during the COVID-19 pandemic [16–20].

Prior to the start of the pandemic in 2020, national reports indicated that hospital visits via the emergency department [21], as well as staff burnout [22], were already on the rise in Canada [23] . In fact, Canadian ICUs were operating at close to full capacity in 2016, particularly in large urban academic centers, leaving little flexibility for surge coverage [24, 25]. Demand for ICU beds is expected to increase with an aging population [26, 27]. Notably, depleted human resources (e.g., nursing availability), affected both bed and ventilator use in the ICU at this time [28]. Existing levels of resource strain have been further exacerbated since the start of the COVID-19 pandemic. High patient volumes and overwhelmed supply chains have led to both national and global shortages of the resources required to care for critically ill patients (e.g. ICU beds, ventilators, staff, etc) [29–31] and to protect critical care providers from contagion (e.g. medical masks, N95 respirators) [32]. In extreme cases, insufficient resources have resulted in triaging life saving interventions (e.g. mechanical ventilation) [33] and unduly exposing providers to the virus [34–36]. Risk of viral transmission increases when performing aerosol generating medical procedures (AGMP) [37], particularly tracheal intubation, which is commonly performed by frontline critical care providers [38, 39]. Executed without adequate personal protective equipment (PPE)—gloves, gowns, eye protection, N95 respirators—may further compromise frontline provider safety [40]. It remains unclear how anticipated or actual resource shortages have affected critical care physicians and the delivery of patient care in ICUs in Canada during the pandemic. To address this gap in our knowledge, and to generate information that may be used to inform hospital and provider focused guidance, we sought physicians’ perceptions and experiences of caring for critically ill patients in the context of actual or anticipated resource strain across multiple institutions during the COVID-19 pandemic.

## Methods

### Study design

We used a qualitative description study design [41] conducted in accordance with the Consolidated Criteria for Reporting Qualitative Research. A qualitative research design is appropriate to explore topics where little information is previously known and to provide in depth information of participants’ own meanings and experiences. The use of qualitative inquiry offered the unique opportunity to develop rich insight into critical care physicians’ perceptions and experiences of resource strain in the ICU during the COVID-19 pandemic. The Research Ethics Boards at the University of Calgary (#REB20–0377) and Dalhousie University (# REB2020–5106) approved this study. Participants provided explicit oral consent in lieu of written consent.

### Participants

We recruited critical care physicians using a recent and accessible sampling frame previously developed by our team [42] of all clinical and academic faculty from adult critical care training programs in 13 Canadian universities. We used non-probability purposive sampling [41] to invite a diverse sample of female and male physicians, currently employed, across years of practice, in provinces representing three (Atlantic, Central, Prairies) of Canada’s five [Atlantic, Central, Prairies, Pacific, Northern Territories] major regions to ensure variation in experiences. We aimed to recruit 5–8 (15–24 in total) participants per region. To this end, we consecutively emailed invitations until we achieved data saturation [43]. No additional eligibility criteria were applied.

### Data collection

We iteratively developed a semi-structured interview guide and pilot tested it with two critical care physicians to ensure the core questions and probes adequately addressed targeted topics. In the pilot test of the guide, we removed one question that asked if the participants’ ICU had admitted any patients with a diagnosis of COVID-19. We also added a question as suggested by one pilot participant in the interview de-brief around the ethical and legal parameters of working under resource shortages in a pandemic. Interview questions asked physicians to reflect on three major topics: (1) current capacity of their primary ICU to meet the needs of critically ill patients with COVID-19, (2) existing or anticipated factors that would jeopardize physicians’ ability to manage the care of COVID-19 patients effectively, and (3) implications for the healthcare workforce and the delivery of patient care (Additional file 1). Interview questions were developed from topics of interest in the news media, clinical discussions and validated through discussion with ICU colleagues [44–46]. We included demographic questions at the end of the interview guide to capture participant age, sex, marital status, clinical base specialty, and ICU size.

Interviews and pilot tests were conducted in English via telephone in a private office by two senior research associates (LK, CdD), both female and with Masters degree training in qualitative and health services research. Participants provided informed verbal consent prior to participating. Interviews were collected between March 25 to June 25th, 2020. We digitally recorded the interviews and sent the audio files to a transcription company (www.rev.com/) to produce verbatim transcripts. The interviewers (LK, CdG) reviewed, cleaned, and de-identified the textual data prior to analysis. Participants were offered the opportunity to review their interview transcripts as a form of member checking to maximize validity.

### Data management and analysis

Qualitative analysis was conducted between April 27 and July 25, 2020. We used NVivo 12 (https://www.qsrinternational.com/) to manage the data and facilitate thematic analysis. Two researchers (LK, CdG) first reviewed and coded a small sample of the transcripts (*n* = 3) independently and in duplicate using open coding [47]. Initial codes were compared and discussed with a senior qualitative researcher (JPL) to create a first draft of the codebook. The researchers then analyzed an additional three transcripts using both open and axial coding, iteratively refining the codebook until all relevant ideas were included. Deviant cases and exceptions within the data were sought and resulted in alterations to the codebook if the data were relevant to the research question, interview questions and emerging themes. The complete dataset (*n* = 15 transcripts) was then coded in duplicate with the finalized codebook. Fracturing of the data through axial coding was particularly useful in theme development as it enabled researchers to look across interviews for nuanced differences in participant perceptions. Both researchers used memos to document initial ideas during interviews as well as to document the relationships between codes during analysis. The researchers held weekly meetings during the 4-month period of analysis, wherein themes were developed, revised, and refined. Although our team was prepared to recruit further, saturation of overarching themes was achieved after the codebook was stabilized but prior to full analysis of the dataset (approximately halfway through the 4-month data analysis period).

## Results

We contacted 44 potential participants of which we interviewed 15. Non-respondents were contacted once following the initial email invitation. We conducted all interviews between March 25 and June 25, 2020. Interviews lasted a median (IQR) of 21 (15.3, 26.1) minutes. Table 1 presents the participant characteristics. The participants worked across 14 different ICU’s. All but one participant reported that patients with a diagnosis of COVID-19 had been admitted to their ICU at the time of interview. One participant offered to participate in member checking of their transcript.

**Table 1:** Interview participant characteristics

Characteristics	Participants (*n* = 15)
Institution Type*, n (%)*	
Tertiary	13 (87)
Community	2 (13)
Age, median (IQR)	40 (38–47)
Female, *n* (%)	8 (53.3)
Marital status, *n* (%)	
Single	3 (20)
Married/Common Law	12 (80)
Has children, *n* (%)	10 (66.7)
Secured living accommodations away from children	2 (13.3)
Years since finishing residency, median (IQR)	9 (5–16)
Base specialty, *n* (%)	
Internal Medicine	7 (46.7)
General Surgery	3 (20)
Anaesthesiology	3 (20)
Other^a^	2 (13.3)
Province, *n* (%)	
Alberta	5 (33.3)
Ontario	5 (33.3)
Quebec	3 (20)
Nova Scotia	2 (13.3)

^a^other base specialties included pediatrics and respirology

**Fig. 1:**
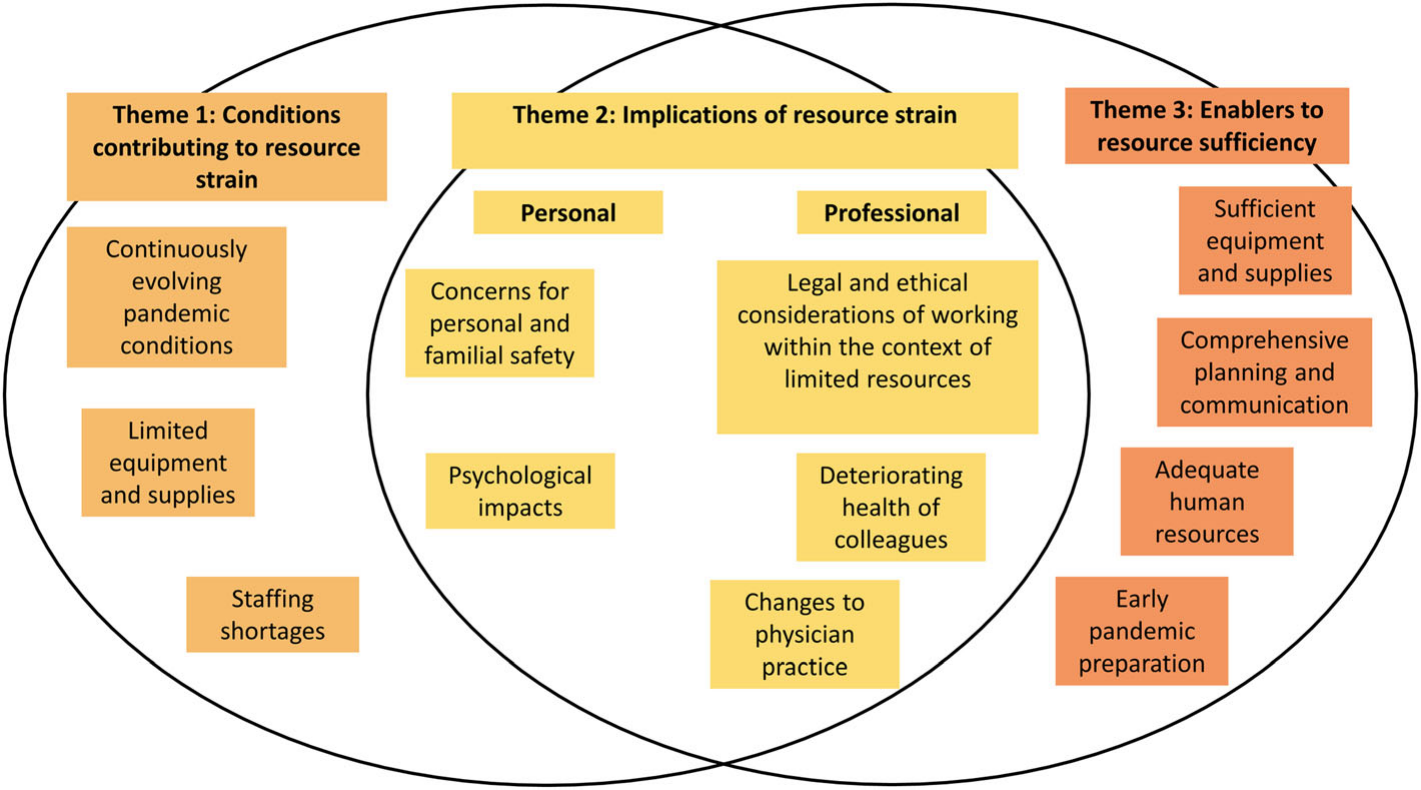
Visual representation of themes and subthemes describing critical care physicians’ perceptions and experiences of caring for patients in the context of resource strain during the COVID-19 pandemic

Analysis revealed three overarching themes and multiple associated subthemes. Overarching themes included: 1) Conditions contributing to resource strain (e.g., continuously evolving pandemic conditions), 2) Implications of resource strain on critical care physicians personally (e.g., safety concerns) and professionally (e.g., practice change), and 3) Enablers of resource sufficiency (e.g., adequate human resources) (Fig. 1).

Overarching themes represent ideas that were largely echoed and emphasized by all participants in the study, while subthemes illustrate unique ideas related to a primary theme and provide a window into the diversity of participants’ perceptions and experiences. Subthemes were also shaped by the variability in pandemic preparations and COVID-19 case burden across ICUs and geographic regions. Quotations that exemplify themes and sub-themes are included in Tables 2, 3 and 4.

**Table 2:** Subthemes theme 1, Conditions contributing to resource strain descriptions and quotes, Participant ID_SexAge_Region_InstitutionType

Subtheme	Quote
*1. Limited equipment and supplies (supply chain shortages)* Lack of equipment, supplies and space needed to care for ICU patients. Could be due to supply chain shortages.	*“Our supply chain is already threatened and some organizations are always within 24, 48 h of running out [of PPE]. I know of a hospital in our region that went to Walmart to buy some surgical masks on Sunday. So they’re always a threat with running it within 24, 48 h.”* – Participant 5_M60_Central_ Tertiary
*2. Staffing shortages* Staffing shortages potentially due to HCP illness	*“There’s a lot of planning that’s going on right now. I think there are excellent plans in place, I just am not sure about implementation, and by that I mean it’s really hard to predict the numbers, when we’re going to have to implement the various phases, and also are we going to have sufficient staff to care for all these patients?”*- Participant 4_F49_Central_Tertiary *“Another thing, if people are feeling overworked or distressed, then the potential for moral distress and burnout and empathic distress, all of those things would impact people’s capacity to be truly present for their patients and maybe even not able to come into work for psychological reasons as well. So I think both the physical and the psychological aspects of not being supported adequately would be important factors that could contribute.”* – Participant 11_F40_Prairies_Tertiary
*3. Continuously evolving pandemic conditions* The nature the continuously changing environment makes it difficult to predict what is coming.	*“I’m really hoping that this is something that we’ve planned well for, prepared for, and it never really becomes as bad as we’re all worried that it might be. So far numbers seem good, but that can change on a daily basis.”*- Participant 8_M38_Prairies_ Tertiary *“The things that would jeopardize that would be the frequency of patients that require admission to ICU, overwhelming our current ability to provide that. So in other words, if too many patients come in all at the same time, then we would potentially be overwhelmed. So that would be likely the biggest threat. In contrast to that, if the patients come in well spread out, even if there’s a lot of them, we can cope quite easily.”* – Participant 2_M40_Prairies_ Tertiary

**Table 3:** Subthemes for theme 2, Implications of resource strain descriptions and quotes, Participant ID_SexAge_Region_InstitutionType

**Personal subthemes**	**Quote**
*1. Psychological impacts* Fear, anxiety and other emotions related to working during a pandemic	“*It’s not necessarily a logical fear. It’s fear because of fear. In some sense, we’ve talked about this anticipation of this terrible thing coming has been more fear generating than if we just had a whole bunch of patients and we’re working with them. Because then you’d be so busy working, you’d forget. Perhaps the fear will be put aside to a certain extent.”* – Participant 5_M60_Central_ Tertiary
*2. Concerns for personal and familial safety (of oneself and their family)* Potential to be exposed to patients who are contagious and the added risk that anybody you interact with may be infected were an implication as was changes to daily routines to minimize transmission of the virus	*“The worst-case scenario is that you’re put in a position where you’re interacting with patients who are contagious, are infected, are contagious, and you’re potentially going to get it. And this has personal implications all along the line. Anybody can be infected. Anybody can get seriously ill and die from it, so that is a risk. And then of course if you do, then you’re perhaps unwittingly taking it home and spreading it to other members of the household and family members. So that is always in the background.”* – Participant 5_M50_Central_ Tertiary *“I guess with PPE shortages in place, I think I’ve changed some of my behaviors when I’m working, things that I haven’t really done before. Like I wash my hair every single day now and I put everything in the laundry like right after I come back from work and stuff that I wouldn’t have really thought of doing before.”* – Participant 12_F35_Prairies_ Tertiary *“I think a lot of the distress that we have or I certainly have had is the waiting. […*] *Waiting for the disaster to happen. It’s like a minute-to-minute assessment of the count and the situation and the admissions because I just don’t know when the next shoe is going to drop, and when I’m the one that’s there on the front lines, having to deal with it is quite anxiety-provoking. I almost feel like I just want it to come so then we can just get started*.” – Participant 4_F49_Central_ Tertiary
**Professional subthemes**	**Quote**
*1. Deteriorating health of colleagues* As colleagues get sick the size of the healthcare team reduces.	***“****If we start getting healthcare worker getting infected for example the intensivists then we will have less physician working in the ICU and then pushing our leads that have to work more in the ICU.”* – Participant 7_F44_Central_Community
*2. Changes to physician practice* Practice changes to reduce the amount of PPE used	***“****If we don’t have adequate PPE then I’m not going to be as good a physician for my patients because I’m going to be scared to go into the rooms and less likely to go into the rooms, and the patients won’t get the attention that they would otherwise have gotten.”* – Participant 9_F42_Atlantic_ Tertiary *“There’s been strategies to, if a room is in isolation, limit the numbers of in and outs. So grouping tasks so that they can be done together, grouping medication administration so done together, reducing the number of times medications are administered if possible to reduce the number of times in and out. The cohorting of activities and people to minimize the use of PPE”* – Participant 5_M60_Central_ Tertiary *“Usually I must have 50% of my time protected for research, but now with this pandemic there’s no protected time for that. So I’m doing more clinical work than I should do. So I think everything that is not related to clinical activity has been pushed aside for a while.”* – Participant 6_M40_Central_ Tertiary *“One thing we’ve done to try to preserve PPE is that COVID patients who are in a negative pressure room who are not intubated, or even if they’re intubated, if they’re awake and have a cell phone, we communicate with them by texting. So, if there’s no urgency to go in, we can ask some questions from outside the room and they can answer us, or if they need something they can text us. So, it’s to reduce the number of times people have to go into the room and use PPE and expose themselves.”* – Participant 15_F56_Central_ Tertiary
*3. Legal and ethical considerations of working within the context of limited resources* Conversations around legal protection, resource allocation and expectations for physicians working during a pandemic	***“****I am worried because the [governing college of physicians] said that they would not support us if we were not going to give care to patients because we don’t have the protection. So that worries me a lot. I don’t think we should go and blindly fight for a disease if we’re not protected adequately.”* – Participant 3_F32_Central_ Tertiary “*What we were told [by our governing body] was that the hospital is responsible to its employees but not to the physicians because we’re not employees. And so a nurse could certainly say, “I’m not doing this and not come to work if there was inadequate PPE”. But as physicians, as contract workers, where our Hippocratic oath says otherwise, we probably would have a harder time defending it.”*– Participant 9_F42_Atlantic_ Tertiary *“There’s really no labor law for physicians. Literally, I don’t think that there’s any protection for physicians when it comes to not wanting to take care of patient because of a lack of PPE. That being said, we did very briefly bring that up, but none of us really could deny care. When we have a lack of PPE, I think morally and ethically, I don’t think that we would do that” –* Participant 7_F44_Central_Community *“The major impact [a PPE shortage] would have for me professionally that I would be caught between prioritizing my own safety and taking care of my patients. And that’s a dilemma I actually don’t want to be in. So, that would cause a fair bit of stress and anxiety if we reached that level.”* – Participant 10_M47_Atlantic_ Tertiary

**Table 4:** Subthemes for theme 3, Enablers to resource sufficiency descriptions and quotes, Participant ID_SexAge_Region_InstitutionType

Subtheme	Quote
*1. Sufficient equipment and supplies* Resources such as ventilators, staff, space and single use resources such as PPE and medication	“… *You need the physical supplies that would allow healthcare providers to safely be in the room with somebody who’s got some sort of novel viral pathogen. So, that would be like adequate PPE.”* – Participant 9_F42_Atlantic_ Tertiary *“There’s infrastructure, so there’s places to care for these patients if they grow to a volume that’s greater than our current ICU capacity, which is what’s expected. In addition to that there’s ventilators that are required to ventilate the people that [have] the most severe form of the disease.” –* Participant 1_M40_Prairies_ Tertiary *“The most publicized one would be the ventilators, but even also things like monitors and IV pumps are the ones that come to mind and even the physical [ICU] beds”* – Participant 12_F35_Prairies_ Tertiary
*2. Comprehensive planning and communication* Planning and communication in response to optimizing resource supplies including PPE guidelines vary across institutions in messaging, clarity, degree of protection and excessiveness	*“I do think that had we been in certain country or certain cities in the state, we’d have been in much bigger trouble. But although at the beginning, yeah, we had nothing. Zippo, zilch. There was nothing learned from SARS in our region. There was no plan. I think that’s it*.” – Participant 9_F42_Atlantic_ Tertiary *Initially the recommendations were that we didn’t have to wear masks in the hospital and now it’s changed that everybody has to wear their mask. It changed from enhanced PPE for certain procedures, whereas before it was just regular PPE. So they’ve increased the amount or the level of protection as we’ve gone on from even last week, and I think that’s in response to health care workers demanding that for their own safety”* – Participant 4_F49_Central_ Tertiary **“***The tubing is heavy and sometimes the connections just come apart. And so in order to protect the health care providers, once you are caring for a patient who has known or suspected COVID, then you need to be in your N95 in that room all the time […] which is tricky because we do have a shortage of appropriate PPE.”* – Participant 9_F42_Atlantic_ Tertiary *“for a physician or a nurse who was going in [to the patient room] for maybe just one or two minutes just to check on something briefly with the patient, whether we need to have full PPE on if they’re within a closed circuit, I do question that as opposed to just using the usual precautions for patients who have COVID and are not undergoing an aerosol generating medical procedure. So yeah, I have questioned that.”* – Participant 11_F40_Prairies_ Tertiary
*3. Adequate human resources* Includes factors that support different health care providers, hospital staff, family members and the communication between the different stakeholders in service of resource sufficiency	*“People talk about, the number of ventilators that we have, how many patients we could have to ventilate, and they even talk about beds. It doesn’t really matter if you don’t have the people to manage the patients in those beds.”* – Participant 9_F49_Central_ Tertiary “*The most important things are going to be obviously staff, so that’s going to include physicians, nurses, RTs, and then all the allied health care workers that includes the people that clean the rooms, nursing aids, dieticians.”* – Participant 2_F40_Prairies_Tertiary
*4. Early pandemic preparation* Early preparation on the part of the institution, unit, leadership or individual physicians to prepare for potential surges in patients with COVID-19	*“Our ICU has been very proactive in terms of being able to gather different types of PPE that would replace the PPE that we would use in a normal setting. I’ll give an example. The N95 masks were on a very, very severe shortage, almost like a week left of some of the masks that were available. So our ICU plus the hospital ended up buying respirators, which are the N100s. They’re the industrial ones that people use for painting or welding, working with asbestos and stuff like that. So they actually procured somewhere between 50 and 100 masks for all personnel to use in the ICU. We all get our own filters basically. So that was great.” – Participant 13*_M36_Central_Community

### Conditions contributing to resource strain

Several participants described their ICU as experiencing resource strain (e.g., limited essential supplies) at the time of interviewing, while others felt that strain would occur imminently if the number of patients requiring ICU services continued to rapidly increase in their region. Participants described several factors contributing to strain in their ICU, including: 1) Continuously evolving pandemic conditions (e.g., varying resource supply and demand, unpredictable patient surges), 2) Limited equipment (e.g., rationing of PPE, inadequate physical space in the unit) and supplies (e.g., medication), and 3) Staffing shortages (e.g. physician illness, increased patient demand). Of note, a small group of participants were becoming increasingly concerned about the resource availability in their ICU as requests to accept transfer patients from ICUs that had already reached capacity began to increase (Table 3). Other participants were particularly concerned about the possibility of having to admit multiple patients simultaneously (i.e., in the case of a long-term care home outbreak) and the demand this would place on their ICU and existing pool of limited resources (Table 2). As demonstrated in these examples, participants often framed their discussions of resource strain in the context of uncertainty about whether their ICU would be able to meet rapidly increasing demands as the pandemic progressed. Overall, participants were fairly confident in the pandemic planning occurring in their ICU yet remained unsure as to whether this planning would lead to actual preparedness for what was to come (Table 2).

### Implications of resource strain

All participants in our study described both the personal and professional implications of working in the context of resource strain during the COVID-19 pandemic. In particular, two main subthemes related to the personal toll of their work emerged from interviews: 1) Concerns for personal and familial safety (e.g., transmitting the virus to family members), and 2) Psychological impacts (e.g., increased anxiety). Most striking was the degree of concern for the safety of family members (Table 3). For example, some participants made the difficult decision to self-isolate away from their immediate family members by residing in a hotel to ensure that they would not bring the virus home, while others took great pains to limit their risk of being a contagion by segmenting their home into “hot and cold zones” (i.e., hot zones being the spaces and surfaces that the physician in the family would inhabit or touch, cold zones being those designated to family members). Although the level of concern regarding transmitting the virus to family members did vary across participants from mild to serious, all participants indicated that if they had to work without appropriate PPE they would be very concerned about familial transmission. The unpredictable trajectory of the pandemic had a psychological impact on participants. Feelings of uncertainty about how the pandemic would develop and whether their unit’s planning and supplies would be sufficient, created increased anxiety in many participants as they “*waited for the disaster to happen*”. – Participant 4.

In addition to the noted personal implications of caring for critically ill patients in the context of pandemic resource strain, we identified three subthemes reflecting perceived implications to the healthcare workforce and delivery of patient care (i.e., professional implications):: 1) Changes to physician practice (e.g., less direct patient contact, more clinical work than normal, innovative solutions to PPE shortages), 2) Deteriorating health of colleagues (e.g., decline in colleagues’ physical and/or mental health), and 3) Legal and ethical considerations of working within resource scarcity (e.g., moral dilemma of acting versus not acting). In particular, participants from all regions described changes to their normal clinical practice, specifically emphasizing less direct patient contact as hospitals shifted protocols to minimize the number of people entering the rooms of patients with known or suspected COVID-19 (Table 3).

Several participants also described spending more time than usual on clinical service, while others expressed worrying more about colleagues who they believed were at a high risk due to pre-existing conditions. When asked about legal and ethical considerations of working during a pandemic without adequate PPE, some participants mentioned that their unit had not had any conversations regarding the legal requirements, while others had discussed the matter, yet remained unsure of their legal responsibilities (Table 3). Some participants further investigated their legal obligations by contacting their professional governing body.

### Enablers of resource sufficiency

All participants in this study identified factors that supported individual physicians and ICUs to effectively manage the care of critically ill patients with COVID-19. We clustered enablers into four interrelated subthemes: 1) Sufficient equipment and supplies (e.g., staff, ventilators, unit space, medication), 2) Comprehensive planning and communication (e.g., early pandemic preparation, clear and consistent PPE guidelines), 3) Adequate human resources (e.g., colleagues and personnel), and 4) Early pandemic preparation (e.g., surge planning) (Table 2).

Early pandemic preparation was a particularly salient factor to support resource sufficiency. Participants described several actions that their ICU leadership began executing early in the pandemic to plan for different surge and strain scenarios, such as, updated ICU plans (e.g., creating more ICU beds within and across hospitals), modified call schedules (e.g., creating backup call schedules), and redeployed staff from other units to the ICU to meet increased capacity demands. In addition, all participants mentioned that their units were considering solutions to mitigate and prepare for potential PPE shortages, including recycling, reusing and finding non-traditional PPE alternatives (e.g. painter’s masks, 3D printing face shields).

Similarly, participants across all regions indicated that the early implementation of clear and concise PPE guidelines was a crucial component of pandemic preparedness. Many also noted that while guidelines may need to shift in accordance with pandemic circumstances, keeping staff apprised of these changes in a clear and transparent manner (i.e., rapid knowledge mobilization and translation) was vital. At the same time, although there was consensus across our participant pool regarding the need for clear and concise COVID-19 guidelines, perspectives on what should be included in those guidelines to sustain high quality care and staff safety were more nuanced. Varied opinions were most noticeable in discussing what PPE should be used when entering the room of a patient with COVID-19. For example, participants generally agreed that full PPE (e.g., with N95 respirator) is needed in a closed-circuit intubated patient room because, as explained by Participant 11, *“[there is] risk that at some point, the circuit becomes disconnected spontaneously, exposing everyone in the room [to the virus]”*, but diverged on whether or not full PPE was necessary when entering the room of every patient confirmed to have COVID-19, regardless of medical procedure (Table 4).

## Discussion

Caring for critically ill patients with a known or suspected novel infectious disease during a global pandemic is a complex task that requires multi-level (e.g., organizational, departmental, personal) planning and preparedness [48] to protect patients and health care workers [49]. The availability of resources is clearly an important mediator in successfully managing the care of critically ill patients in pandemic conditions [50]. Research during previous infectious disease outbreaks has shown that ample supplies of PPE significantly facilitated effective clinical care [51, 52], while insufficient or rapidly depleting PPE contributed to healthcare worker anxiety [53]. We conducted semi-structured interviews with critical care physicians in Canada to better understand their perceptions and experiences of caring for critically ill patients in the context of actual or anticipated resource strain during the COVID-19 pandemic. We identified three primary and interrelated themes: conditions contributing to resource strain, implications of resource strain on critical care physicians personally and professionally, and enablers of resource sufficiency.

When considering the management of essential resources required to care for patients with COVID-19, academic and mainstream media sources have been particularly concerned about adequate supplies of equipment (i.e., ventilators) and possible PPE shortages [29, 54, 55]. Our participants reflected similar concerns. In particular, the most salient and worrisome conditions that participants noted involved circumstances outside of their control (e.g., patient surges, supply chain shortages, etc.). At the same time, key enablers to support resource sufficiency in our study consisted of comprehensive planning and communication as well as early preparation. The issue of resource strain is likely further exacerbated by a healthcare system that already faced a growing demand for acute care with an aging population demographic [56]. Some participants described that their units had developed and enacted detailed plans to prepare for surge scenarios, including a tiered response of staffing and capacity depending on the number of patients with COVID-19 admitted to hospital. Other participants felt that their units were less prepared in that they were already experiencing shortages of PPE and were being presented with measures and mandates by their institutions to ration and conserve remaining supplies.

In addition to echoing the importance of adequate material resources, several of our participants also stressed the need for units to ensure that adequate numbers of properly trained personnel were available to care for incoming critically ill patients with COVID-19. In this respect, of particular concern to our participants were occurrences of large outbreaks at long term care facilities and outbreaks in neighbouring cities that would require hospitalization in their unit. Participants carried reports of global surges (where ICU bed need exceeded capacity leading to resource shortages and triage scenarios) [29] at the back of their minds, and largely did not know how their ICU’s pandemic planning would hold up in a similar scenario.

Our findings shed light on the importance of establishing ethical and legal parameters for healthcare workers as part of pandemic planning and preparedness [57]. In Canada, when a physician agrees to treat a patient, they have a legal duty to provide a certain standard of care [58]. In a state of emergency, individual provincial legislation may permit the provincial government to mandate physicians to perform certain services [59]. Yet, Canadian labor boards have outlined criteria to justify refusal to work which may be applicable during a pandemic [60]. Our participants highlighted the legal uncertainty surrounding physicians’ rights and obligations to continue to practice during a pandemic [59]. Some participants had conversations within their unit while others reached out to their governing bodies for guidance regarding the expectations of working as a physician during a pandemic and potentially without adequate PPE. This variability in awareness of the legal and ethical parameters for working during a pandemic highlights national discrepancies and suggests there are inadequate guidelines in Canada for what is expected of physicians during an infectious disease outbreak [59].

It is clear that the impacts of working during an infectious disease outbreak—particularly under conditions of real or anticipated resource strain—extend beyond the workplace setting. A recent systematic review highlighted the burden of mental health symptoms including anxiety, acute stress, depression and burnout among frontline healthcare workers during and following a disease outbreak [61]. Participants in our study experienced varying degrees of anxiety, particularly with respect to concerns for their family [62]. A small number of participants responded similarly to frontline workers around the world by finding alternate living accommodations (e.g., living in a hotel) to minimize exposure and risk to their families [63]. Other qualitative work indicates frontline healthcare workers in the China and the United States also dealt with anxiety about becoming infected themselves or family members becoming sick with COVID-19 [64, 65]. Other participants were much less concerned for the safety of their families, particularly their young children, potentially due to early reports indicating that the virus may not impact children as severely as adults [66, 67]. A general sense of anxiety and uncertainty was prominent throughout the interviews in regard to the pandemic placing the participants and their organizations in uncharted territory. National and provincial predictive models indicated first wave peaks that would induce resources shortages across the country [68–70]. These models may have positively impacted and motivated early pandemic preparations, yet they may have also contributed to the stress and anxiety of healthcare providers and the public alike. While Canadian ICUs have a similar number of beds to Western European countries [71] a comparison in terms of conditions contributing to and implications of resource strain in ICU during a pandemic warrant further exploration.

There are some limitations that should be considered when interpreting our findings. First, the perspectives shared by our participants may not be transferable across Canada, as the majority of our participants practiced at urban academic institutions. Smaller community, regional or rural centres may have had varying levels of preparation and numbers of patients with COVID-19 leading to differences in perceived or actual resource strain. Second, physicians who felt particularly well prepared or conversely, highly strained, in terms of resource shortages may have been more motivated to volunteer for an interview. We attempted to mitigate this by purposively sampling a diverse group of critical care physicians in provinces that had variable numbers of people affected with COVID-19. This was further supported by using telephone interviews which enabled participation outside of the researchers’ home locations. Historically, interviews have been conducted face-to-face as the frame of social interaction and cues (e.g., body language) fits better within this method [72]. However, there is a growing acceptability [73] to conduct telephone interviews and evidence suggests strengths over face-to-face interviews such as ability to concentrate on voice instead of face, the feeling of not being judged and easier rapport over the phone [74]. The use of multiple interviewers has the risk of generating different data due to different interactions and participant perceptions [75], to mitigate this each interviewer followed the same introductory script and wrote memos following each interview that informed analysis. We also kept the interviewers and analysts consistent between data collection and analysis. Non-probability purposive sampling is often critiqued for being ambiguous and subjective.

## Conclusions

The COVID-19 pandemic has placed many of hospitals and healthcare providers in uncharted territory*.* This study investigated physicians’ perceptions and experiences of caring for critically ill patients in the context of resource strain, and identified contributing conditions, implications, and perceived enablers to resource sufficiency. Continuously evolving pandemic circumstances and a sense of uncertainty expressed by our participants highlight the importance of an organized national pandemic response plan for subsequent waves of COVID-19 and future pandemics.

## Supplementary Information

**Additional file 1.** Semi-structured interview guide.

## Data Availability

The datasets generated and analysed during the current study are not publicly available due to the critical care community being small in Canada and raw interview transcripts when considered as a whole may potentially be identifying. The dataset in the current study are available from the corresponding author on reasonable request.

## Abbreviations

PPE: Personal protective equipment
SARS: Severe acute respiratory syndrome
ICU: Intensive care unit
