# Supplementary material for: A qualitative study of physician perceptions and experiences of caring for critically ill patients in the context of resource strain during the first wave of the COVID-19 pandemic
Source: BMC Health Serv Res. 2021 Apr 22;21:374. doi: 10.1186/s12913-021-06393-5 (PMC8061878; doi:10.1186/s12913-021-06393-5)
Supplement: Supplementary file 1 — Additional file 1. Semi-structured interview guide. [file 12913_2021_6393_MOESM1_ESM.docx]

**Additional File 1. Semi-structured Interview Guide**

Preamble

Research assistant introduces self (name, role, study principal investigator) and the study (review objective).

Initial Engagement

1. Has your ICU admitted any patients with a diagnosis of COVID-19?
   - If so, how many patients with COVID-19 has your ICU managed?
2. What do you think are the most important resources needed for ICUs to safely manage COVID-19 cases?

Topic 1: Resource Shortage

1. How would you CURRENTLY assess your ICU’s capacity to meet and manage the needs of patients and ICU clinicians during the current COVID-19 pandemic? (sufficient resources)
2. What factors might jeopardize an ICU’s capacity to safely manage COVID-19 cases during a pandemic? (conditions leading to resource scarcity)?

- If the informant hasn’t yet mentioned any concerns, elicit with probe:
- Equipment shortages (e.g., ventilators, N95 masks, hospital beds)
- Staffing shortages (as health care workers are exposed to the virus)
- PPE guidelines and training (staff knowledge and adherence for appropriate use)
- Resource scarcity contingency plans are in place and widely known by staff

1. Do you think your assessment [of ICU’s capacity to meet needs] will change over time?

- Why? Why not?

1. Do you think your ICU has clear guidelines for optimal use of Personal Protective Equipment for general care of COVID19 patients?
   - Do you think your institutional PPE guidelines provide sufficient protection from COVID-19?
   - Is the recommended PPE excessive?
   - Do you feel that adequate training in PPE technique has been provided? How is this monitored?
2. Do you trust that your administration has prioritized your safety in making decisions about PPE? Has the evidence been transparently communicated?
   - How safe do you think other ICU staff feel?
   - Do you feel the PPE regimen at your institution is sustainable? If not, what do you think will run out?
     1. Has your unit considered innovative approaches to managing PPE shortages (items made in-house etc, reusing N95 masks)?
3. Have there been conversations within your team on the legal or ethical parameters of working under resource shortages during a pandemic?
   - Since the SARS outbreak, stories of healthcare workers taking PPE from different departments or re-selling PPE have come to light. Have you heard of anything similar with the COVID-19 outbreak?

Topic 2: Direct impact of resource shortages on self

1. Can you talk a little more about how anticipated (or potential) resource shortages could directly impact you professionally?

- Psychological and physician strains, stresses and worries, and fears (Examples)
- long hours; relationship to colleagues
- pressures to work without sufficient resources (e.g., PPE); difficult patient cases; unpredictability of cases and health system strains
- Potential to be exposed

1. Could directly impact you personally or your family?

- Probes: Caught between professional obligations and fears of workplace/home safety
- Are you afraid of bringing the virus home to your family?
- Have you made any changes to your routine, behaviours, or living arrangements in response to this fear?

1. When should healthcare workers who were infected with COVID-19 return to work?
2. Are there coping strategies that you have felt are helpful?

Do you have any final thoughts?

Thank you for participating in our COVID-19 study. Your perspectives and experiences are appreciated.
